# Anomaly detection to predict relapse risk in schizophrenia

**DOI:** 10.1038/s41398-020-01123-7

**Published:** 2021-01-11

**Authors:** Philip Henson, Ryan D’Mello, Aditya Vaidyam, Matcheri Keshavan, John Torous

**Affiliations:** 1 Icahn School of Medicine at Mount Sinai, New York, NY USA; 2 Department of Psychiatry, Beth Israel Deaconess Medical Center, Harvard Medical School, Boston, MA USA; 3 Departments of Psychiatry and Clinical Informatics, Beth Israel Deaconess Medical Center, Harvard Medical School, Boston, MA USA

**Keywords:** Predictive markers, Schizophrenia

## Abstract

The integration of technology in clinical care is growing rapidly and has become especially relevant during the global COVID-19 pandemic. Smartphone-based digital phenotyping, or the use of integrated sensors to identify patterns in behavior and symptomatology, has shown potential in detecting subtle moment-to-moment changes. These changes, often referred to as anomalies, represent significant deviations from an individual’s baseline, may be useful in informing the risk of relapse in serious mental illness. Our investigation of smartphone-based anomaly detection resulted in 89% sensitivity and 75% specificity for predicting relapse in schizophrenia. These results demonstrate the potential of longitudinal collection of real-time behavior and symptomatology via smartphones and the clinical utility of individualized analysis. Future studies are necessary to explore how specificity can be improved, just-in-time adaptive interventions utilized, and clinical integration achieved.

## Introduction

Avoiding relapse in early course schizophrenia (SZ) is a priority of effective care, yet today still remains a challenge. Each episode of relapse is associated not only with immediate personal suffering and increased healthcare costs, but also long-term deterioration of functioning, cognition, and quality of life^1^. Prevention of relapse requires prediction, yet today’s prediction models are ineffective^2^ and even parents and family members of patients noted a central unaddressed need due to “limited confidence in recognizing and coping with relapse”^3^. A reason prediction models are ineffective is that while they identify risk factors associated with relapses such as medication adherence, symptoms of anxiety and depression, and substance abuse, they cannot account for the dynamic nature of these risk factors that will vary across time and environments. Advances in clinical care and translation psychiatry require new dynamic markers for relapse that can both guide care and offer novel targets for biological research. In this study, we examine how technology can help quantify risk and predict relapse toward these goals.

There is increasing evidence that digital phenotyping^4^, often also called personal sensing^5^, and other technology-generated signals captured throughout the course of daily life may predict relapse in SZ. Smartphone digital phenotyping utilizes both ecological momentary assessment (EMA) as well as sensors in the phone like GPS, accelerometer, call/text logs, screen time, and response latency to capture longitudinal digital trajectories in patients’ own environments. In prior research, among a sample of 17 people with SZ, we found that digital phenotyping anomalies around mobility, sociability, and EMA detected two weeks prior to relapse were 71% higher than the rate of anomalies during other time periods^6^ and that anomalies often appeared as pairs with two domains changing together. Recent studies are employing similar methods; for example, the EMPOWER study uses smartphones to capture early warning signs of relapse in SZ compared to personal baselines^7^. A 2020 paper on digital phenotyping in SZ reported how these signals can help predict social functioning, an important predictor of clinical outcomes and relapse, that had previously been challenging to quantify in vivo^8^. Recently, other studies have been using anomaly detection on a variety of data. A 2019 paper employed an anomaly detection method on social media posts from people with SZ to predict relapse over a one-month period with a sensitivity and specificity of 0.71 and 0.38, respectively^9^.

The clinical importance of smartphone-based anomaly detection to predict relapse has been highlighted by the COVID-19 pandemic and the clinical mass adoption of telehealth^10^. While there is strong clinical and research evidence that telehealth is feasible for people with SZ, a recent review noted that because many clinical features in the illness are dependent on nonverbal cues, technology is critical to ensuring optimal care^10^. As more care is delivered remotely, these new smartphone-derived data on patients’ clinical trajectories can support more personal, responsive, and informed care—even if delivered thousands of miles apart^11^.

In this present study, we utilized smartphone digital phenotyping to predict clinical relapse in people with SZ. Building off our prior work which utilized anomalies in mobility, sociability, and EMA^6^, here we sought to include further clinically relevant features, including screen time and cognition. To understand if anomalies detected around relapse are unique to SZ, we included an age-matched control group. We hypothesized that, in line with our prior pilot results, we will detect a higher rate of anomalies leading up to clinical relapse compared to periods without relapse in patients and none in healthy controls (HC). Given the clinical heterogeneity in relapse, we also predicted that different combinations of pairs of digital phenotyping features for each patient would predict relapse with higher sensitivity and specificity compared to a population model.

## Methods

### Data collection

A total of 126 participants were recruited from the greater Boston area. Eighty-three participants had a diagnosis of SZ and 43 were HC. Written informed consent was obtained from all study participants the protocol was approved by both the Beth Israel Deaconess Medical Center as well as the State of Massachusetts Department of Mental Health IRBs. Two open-source research applications were installed on participants’ smartphones, mindLAMP^12^ and Beiwe^4^, to collect EMA and sensor data, respectively. These apps were both self-hosted by our team and can be accessed freely by others seeking to reproduce or replicate this work. Data collection began in August 2018 and continued through June 18, 2020. Participant enrollment ranged from 3 to 12 months. EMA frequency also varied and was either twice each day or five times each week. Surveys administered for EMA included the Patient Health Questionnaire-9 (PHQ-9) for depression and the Generalized Anxiety Disorder-7 (GAD-7) for anxiety as well as questions around self-reported sleep, sociability, and psychosis. Participants were also navigated to a modified Trails A/B cognitive task meant to assess visual attention and task switching called Jewels Trails A/B^13^ at end of each batch of surveys. Paper and pencil scales were administered at baseline and throughout the study, every 1–3 months and included PHQ-9, GAD-7, the positive and negative syndrome scale (PANSS), and the clinical global impression (CGI). Throughout the entire study duration, Beiwe was collecting sensor data in the background including GPS, accelerometer, call/text logs, and screen time.

### Anomaly detection

To detect changes in behavior and symptomatology on a day-to-day basis, the method of anomaly detection was employed on the EMA and sensor data. In accordance with previous implementations of the method^6^, feature groups were created to form the input data. The feature groups were: (1) surveys, (2) mobility (from GPS), (3) sociability (from call/text logs), (4) cognition (from Jewels Trails A and B), (5) screen time, and (6) sleep. A breakdown of features can be found in Table 1. Longitudinal feature data was normalized for each participant to establish a baseline, then simultaneously tested for anomalies, i.e., days where given features deviated significantly from that baseline. Anomaly detection and subsequent analysis were performed in the R programming language (version 3.6.3, https://www.r-project.org/) and we publicly share the method to encourage reproducibility (https://www.notion.so/digitalpsychiatry/Anomaly-Detection-in-R-177a40b5120343fdad1bff6db7632118).

**Table 1:** Listing of features within feature groups.

Survey categories	Mobility features	Sociability features	Cognition features	Screen time features	Sleep features
1. Anxiety	1. Time spent at home	1. Number of outgoing texts	1. Jewels Beta A	1. Screen time	1. Sleep duration
2. Social	2. Distance traveled	2. Total outgoing text length	2. Jewels Beta B	2. Session time	
3. Medication	3. Radius of gyration	3. Texting out-degree		3. Checks	
4. Psychosis	4. Maximum diameter	4. Number of incoming texts			
5. Depression	5. Maximum distance from home	5. Total incoming text length			
6. Sleep	6. Number of significant locations	6. Texting in-degree			
	7. Average flight length	7. Texting reciprocity			
	8. Standard deviation of flight length	8. Texting responsiveness			
	9. Average flight duration	9. Number of outgoing calls			
	10. Standard deviation of flight duration	10. Total outgoing call duration			
	11. Fraction of the day spent stationary	11. Call out-degree			
	12. Significant location entropy	12. Number of incoming calls			
	13. Minutes of GPS data missing	13. Total incoming call durations			
	14. Physical circadian rhythm	14. Call in-degree			
	15. Physical circadian rhythm stratified	15. Call reciprocity			
		16. Call responsiveness			

Features are calculated for each participant for each day data is available. Cognition features are derived from a modified Cox proportional hazard model previously reported on^13^. Screen time features are derived from power state sensor data and checking behavior specifically (phone sessions lasting < 15 s) has been shown to be a proxy for problematic smartphone usage^20^. Sleep duration estimates were generated from sensor fusion of GPS, accelerometer, and screen usage events, by defining a significance threshold for filtration of extraneous observations. For GPS, the Euclidian distance between two events was taken; for accelerometer, the difference in magnitude between two events was taken; for screen usage, the duration of time the screen was turned on or the device was unlocked was taken. These three streams were binned in 24-h intervals starting at 12:00 (noon), and for all three sensors, the median of both sleep time and wake time were taken per bin to create a single sleep period.

### Clinical targets

Relapse was defined a priori as one of the following: (1) psychiatric hospitalization, (2) 25% increase in PANSS from baseline, (3) a CGI change score of 6 or 7 corresponding to “much worse” or “very much worse,” and (4) increase in the level of care or exacerbation symptoms that required immediate clinical management as assessed by the patient’s own personal clinician (ie non-study clinician) through medical records and review with that clinician - after that patient concluded the study protocol. This definition is in line with current definitions^14^ used across the field and also in smartphone app research related to relapse^15^ in the same population. These clinical targets related to relapse were then compared to derived anomalies to determine the sensitivity and specificity of anomaly detection in predicting relapse.

## Results

Of the 126 participants, 26 were excluded for not providing at least 2 weeks of data. An additional 10 controls were excluded for having baseline or follow-up PHQ-9 scores greater than or equal to 5. Of the remaining 90 participants, 63 were individuals with schizophrenia, and 27 were HC (Table 2). The 63 were individuals with schizophrenia contributed a mean of 126 days of data and the 27 HC 90 days of data. Engagement with surveys was 59% for those with schizophrenia and for HC was 53%.

**Table 2:** Ninety individuals from the greater Boston area participated in this study.

	HC (*n* = 27)	SZ (*n* = 63)	*p*
Age	32.48 (16.34)	36.45 (14.96)	0.284
*Gender*			0.739
Female	10 (37.0%)	24 (38.1%)	
Male	14 (51.9%)	35 (55.6%)	
Other	3 (11.1%)	4 (6.3%)	
*Race*			<0.001
American Indian or Alaskan Native	0 (0.0%)	4 (6.6%)	
Asian	19 (70.4%)	1 (1.6%)	
Black or African American	3 (11.1%)	18 (29.5%)	
Multiracial or Other	0 (0.0%)	5 (8.2%)	
White Caucasian	5 (18.5%)	32 (52.5%)	
Native Hawaiian or Pacific Islander	0 (0.0%)	1 (1.6%	
*Education*			<0.001
4-year college graduate or higher	23 (85.2%)	18 (28.6%)	
High school graduate/GED	2 (7.4%)	19 (30.2%)	
Some college	2 (7.4%)	21 (33.3%)	
Some high school	0 (0.0%)	5 (7.9%)	

### Single anomalies

**Fig. 1:**
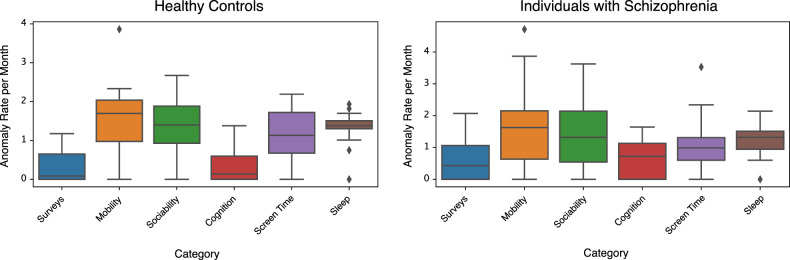
Box and whisker plots of monthly anomaly rates for each feature, separated by diagnostic group. The box edges represent the 25th and 75th percentiles and a median line is drawn within each box. The whiskers extend to 1.5 times the interquartile range in both directions, and outliers are plotted separately.

Overall, 73 participants had at least one anomaly and there was 1006 anomalies total. Monthly anomaly rates for each participant varied from as low as 0 to as high as 4.7 (Mobility). Variation was also seen between anomaly rates of individual features (Fig. 1).

### Paired anomalies

There were 39 participants (28 individuals with schizophrenia and 11 HC) who experienced at least 1 day with paired anomalies. Of the 28 participants with schizophrenia who experienced a day with the paired anomaly, 7 (25%) had survey anomalies combined with a passive data anomaly and the rest had all passive data anomalies. Of the 11 HC who experienced a day with a paired anomaly, 1 (9%) had survey anomalies combined with a passive data anomaly and the rest had all passive data anomalies. The total number of days of paired anomalies per person ranged from 1 to 7 in individuals with schizophrenia (mean = 2.0, sd = 1.3) and from 1 to 5 in HC (mean = 2.5, sd = 1.4). All six feature categories were represented at least once in a paired anomaly. No participants experienced more than two anomalies on a single day. Of note, there were no days with paired anomalies for people with schizophrenia or HC after mid-March 2020 which was when the State of Massachusetts begin to institute social distancing policies around COVID-19. Eleven people with schizophrenia were active in the study during this time period.

### Relapse

Based on our a priori definitions of relapse, there were no participants that were hospitalized, 4 that experienced more than a 25% increase in PANSS, 2 that had a CGI change score greater than or equal to 6, and 23 with noted increased care or significantly exacerbated symptoms based on clinical judgment and/or medical record. There were no relapses during the COVID-19 period of the study.

There were three true relapsed participants according to the above definitions that were not captured by anomaly detection and 48 participants who were correctly identified as non-relapse. Overall, we found that paired anomalies derived from the method had a sensitivity of 89%, a specificity of 75%, a positive predictive value (PPV) of 60%, and a negative predictive value of 94%.

**Fig. 2:**
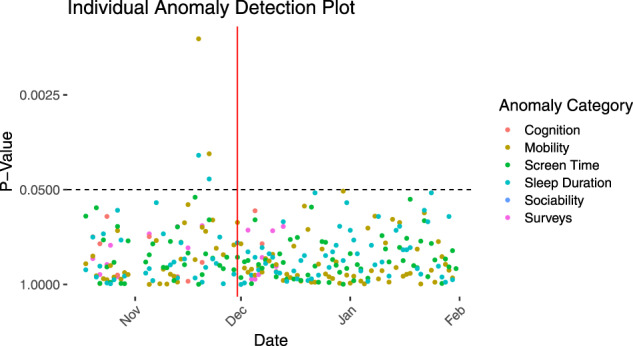
Individual participant plot of single and double anomalies. The *x*-axis represents the date and the *y*-axis, plotted reverse logarithmically, represents the p-value of feature anomalies. The higher up on the *y*-axis, the smaller the *p* value, and the more significant. A horizontal dotted line is drawn at *p* = 0.05. Each dot represents the detection of an anomaly for a particular category on that day, with the dots above the horizontal dotted line considered significant. The red vertical line represents a relapse event (in this case, exacerbation of symptoms). Single anomalies above the significance threshold were hidden for clarity of interpretation.

Longitudinal anomaly plots were generated for each participant as a tool for visualizing the single anomaly pattern throughout the study duration as well as paired anomalies leading up to relapse, when applicable (Fig. 2).

## Discussion

In this study, we used smartphone-captured digital phenotyping data to predict clinical psychotic relapse and found the algorithm to have 89% sensitivity and 75% specificity in our sample of 90 individuals from the greater Boston area. These findings offer relevance for both clinical care as well as translational research.

While the monthly anomaly rates for mobility and sociability were in line with previous research and around the expected value of 1.5^6^, the monthly rate of survey anomalies was significantly lower in both individuals with schizophrenia (0.43) and HC (0.08). One reason for lower rates among the patient group may be due to the fact that there is inherently more incoming data from smartphone sensors that are always on than from EMA, leading to fewer chances for anomalies among surveys. Another reason is that as patients approach relapse and experience changes in cognition, they may report symptoms differently and, in some cases, lose insight into symptoms^16,17^, which further complicates reliance on EMA. Thus, the divergence between active and self-reported EMA data from passive and sensor data may offer a new target itself for relapse prediction and insight for focused therapies. It also offers a new target for biological research and translational psychiatry effort seeking to link genetics, neuroimaging, and physiological to personal patterns of relapse.

Interestingly, despite excluding controls with high baseline clinical scores, over a quarter of the participants with paired anomalies were HC, contributing to a lower PPV of 60%. This could be due to the fact that many of the controls in the study are undergraduate students with variable schedules and academic demands outside those of the general population. It may also reflect the high mental health burden in students and suggest our methods could offer a new means to explore mental health needs in this population. While there were no days of triple anomalies for any participant, every feature category was represented in the paired anomaly results, which suggests that there is a useful signal in each of the data streams. Our study ran for over one year meaning that some participants were active during the holiday season or other time periods that may have been more (or less) triggering. One strength of our method is the ability to collect this longitudinal data and understand how each participants’ unique lived experience may relate to their risk of relapse.

The clinical relapse definitions used in this study are the top four reported definitions for relapse according to a review of 150 publications and guidelines in 2013^18^. These definitions were determined and publicly reported a priori to any data collection. While there were no psychiatric hospitalizations in our study, there were significant changes in clinical scales (PANSS, CGI) in 8 participants and either increase in medication or exacerbated symptoms as noted by a clinician in 23 participants. The lack of hospitalizations may be due to the relative stability of the participants’ symptoms and/or a high level of connection to care. Results for those without care or in settings with fewer resources would likely be different. Still, with early warning and quick clinical intervention, relapse can often be avoided. The unique pairing of anomalies that characterized each patient’s risk support the notion of personalized trajectories of relapse and the utility of using personal sensing technology to quantify this risk.

While this is not a COVID-19 study, we did have data collection for eleven participants ongoing during April through June of the pandemic. We did not find any paired anomalies, or have any record of relapse, for either people with schizophrenia or HC during this time period. This suggests those partaking in this study may have been stable during the early months of COVID-19 and that our method is a practical means to capture clinically relevant data during public health emergencies. However, our results cannot be generalized to the mental health response of people with schizophrenia or HC to COVID-10, as larger, more diverse, and longitudinal samples are required.

The potential of anomaly detection to identify individuals at risk of relapse has important implications for just-in-time adaptive interventions (JITAI)^19^. Smartphones have shown great utility as scalable devices for data collection and most contain the computing power for simultaneous analysis with methods like anomaly detection. These real-time methods can alert a patient or provider of a significant change in behavior or symptomatology before a potential relapse event would occur. While future research is needed to further improve and refine these methods and their smartphone deployment, the potential for JITAI can already be seen. Anomalies can serve as tailoring variables in JITAI, offering real-time and personalized responses ranging from customized self-help resources delivered on the smartphone to activating emergency response plans.

This study has limitations that must be addressed. First, variability in study durations and survey schedules may have affected anomaly rates as some participants were simply enrolled for longer than others and/or provided higher resolution EMA data. Second, smartphones are a proxy for behavior and do not represent the full context of someone’s environment. For example, a phone left on a table for several hours may be incorrectly interpreted as inactivity or sleep. Third, not all definitions of relapse provide a specific date, making identification of a potential window for intervention difficult. For example, hospitalizations and clinical visits to address exacerbation of symptoms are usually associated with discreet dates, whereas changes in PANSS or CGI over a period of months do not. Finally, HC was not matched for age and race, potentially introducing confounding variables. As noted above, the control group appeared to have higher levels of psychopathology detected by anomaly detection detected by clinical screening.

Our results show the potential of using longitudinal sensor data to inform relapse risk. The strengths of this approach are the scalability of data collection and analytical methods, the individualized analysis that allows for relapse risk to be calculated at a personal level, and the ability for smartphones to be used both for data collection and a point of intervention in the future.

## Conclusion

Smartphone-based anomaly detection offers a feasible method to inform relapse risk in serious mental illness. Our results of 89% sensitivity and 75% specificity in predicting relapse in schizophrenia demonstrate the potential of longitudinal collection of real-time behavior and symptomatology via smartphones and the clinical utility of individualized analysis. Future studies are necessary to explore how specificity can be improved, JITAIs utilized, and clinical integration achieved.
